# Supplementary material for: The neoepitope of the complement C5b-9 Membrane Attack Complex is formed by proximity of adjacent ancillary regions of C9
Source: Commun Biol. 2023 Jan 13;6:42. doi: 10.1038/s42003-023-04431-y (PMC9838529; doi:10.1038/s42003-023-04431-y)
Supplement: Supplementary file 2 — Supplemental Material [file 42003_2023_4431_MOESM2_ESM.pdf]

SUPPLEMENTARY INFORMATION

The neoepitope of the complement C5b-9 Membrane Attack Complex is formed by proximity of adjacent ancillary regions of C9

Charles Bayly-Jones*[1,2], Bill H.T. Ho*[1], Corinna Lau[3], Eleanor W.W. Leung[1], Laura D'Andrea[1], Christopher J. Lupton[1], Susan M. Ekkel[1], Hariprasad Venugopal[4], James C. Whisstock[1,2,5], Tom E. Mollnes[3,6,7], Bradley A. Spicer[1,2#], Michelle A. Dunstone[1#]

[1]Biomedicine Discovery Institute, Department of Biochemistry and Molecular Biology, Monash University, Melbourne, Australia.
[2]ARC Centre of Excellence in Advanced Molecular Imaging, Monash University, Melbourne, Australia.
[3]Research laboratory, Nordland Hospital Trust, Bodø, Norway.
[4]Ramaciotti Centre for Cryo-Electron Microscopy, Monash University, Clayton 3800, Victoria, Australia
[5]EMBL Australia, Monash University, Melbourne, VIC, 3800, Australia.
[6]Institute of Immunology, Oslo University Hospital and University of Oslo, Oslo, Norway.
[7]Centre of Molecular Inflammation Research, Norwegian University of Science and Technology, Trondheim, Norway.

*These authors contributed equally
#Correspondence to bradley.spicer@monash.edu or michelle.dunstone@monash.edu

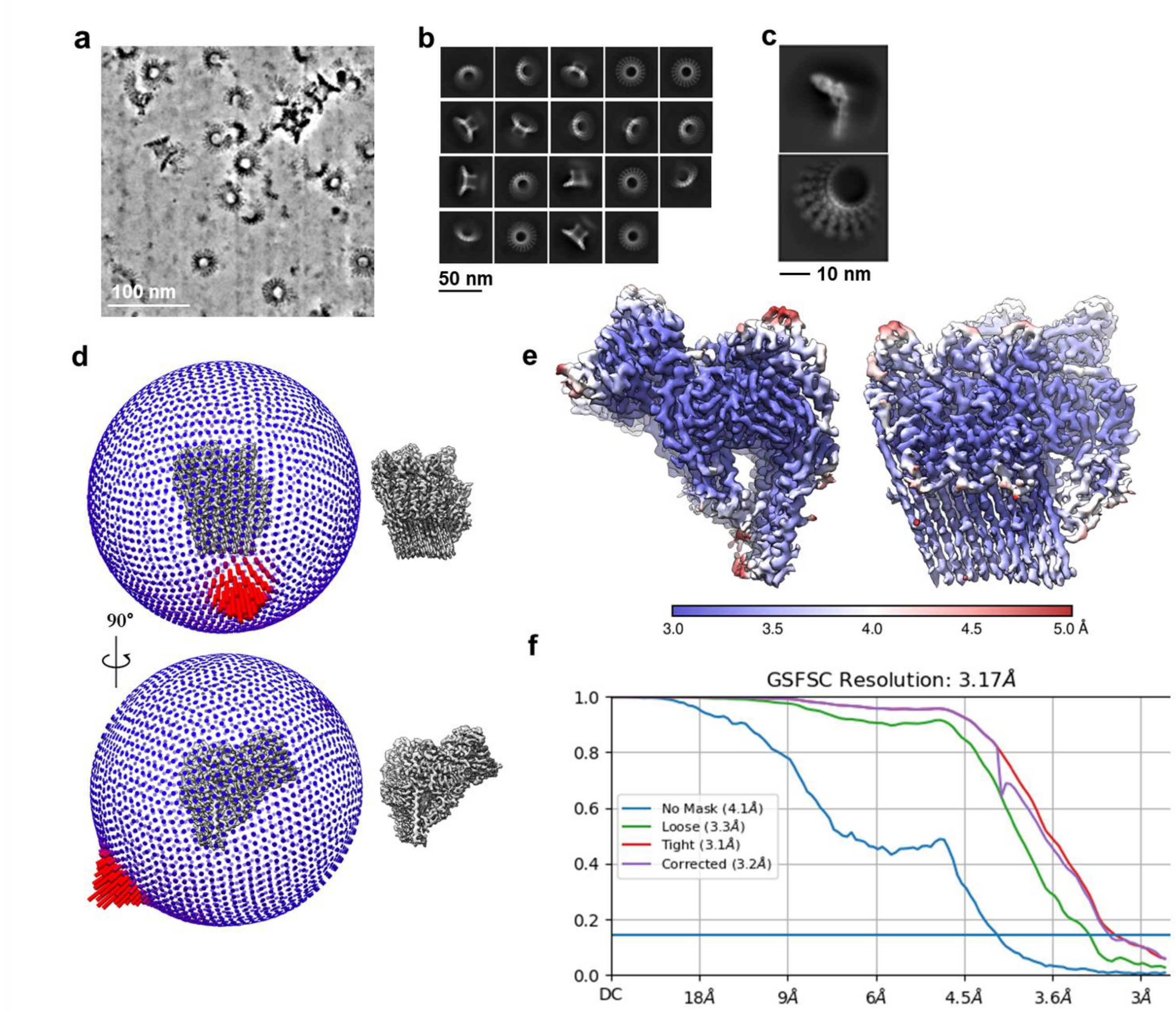

Supp Figure 1. Electron microscopy of polyC9 in complex with aE11-Fab. a. Raw denoised cryo-electron micrograph of polyC9 in complex with aE11. **b.** Two-dimensional class averages of whole aE11-Fab/polyC9 complex. **c.** Sub-particle 2D class averages of localised segment of the aE11-Fab/polyC9 complex. **d.** Angular distribution of viewing orientations and corresponding isosurface rendering of the final reconstruction. **e.** Local resolution isosurface rendering showing centrally located high resolution features, including the aE11-Fab/polyC9 binding interface. **f.** Gold-standard Fourier shell correlation (GSFSC) plot and estimate of (masked) global resolution.

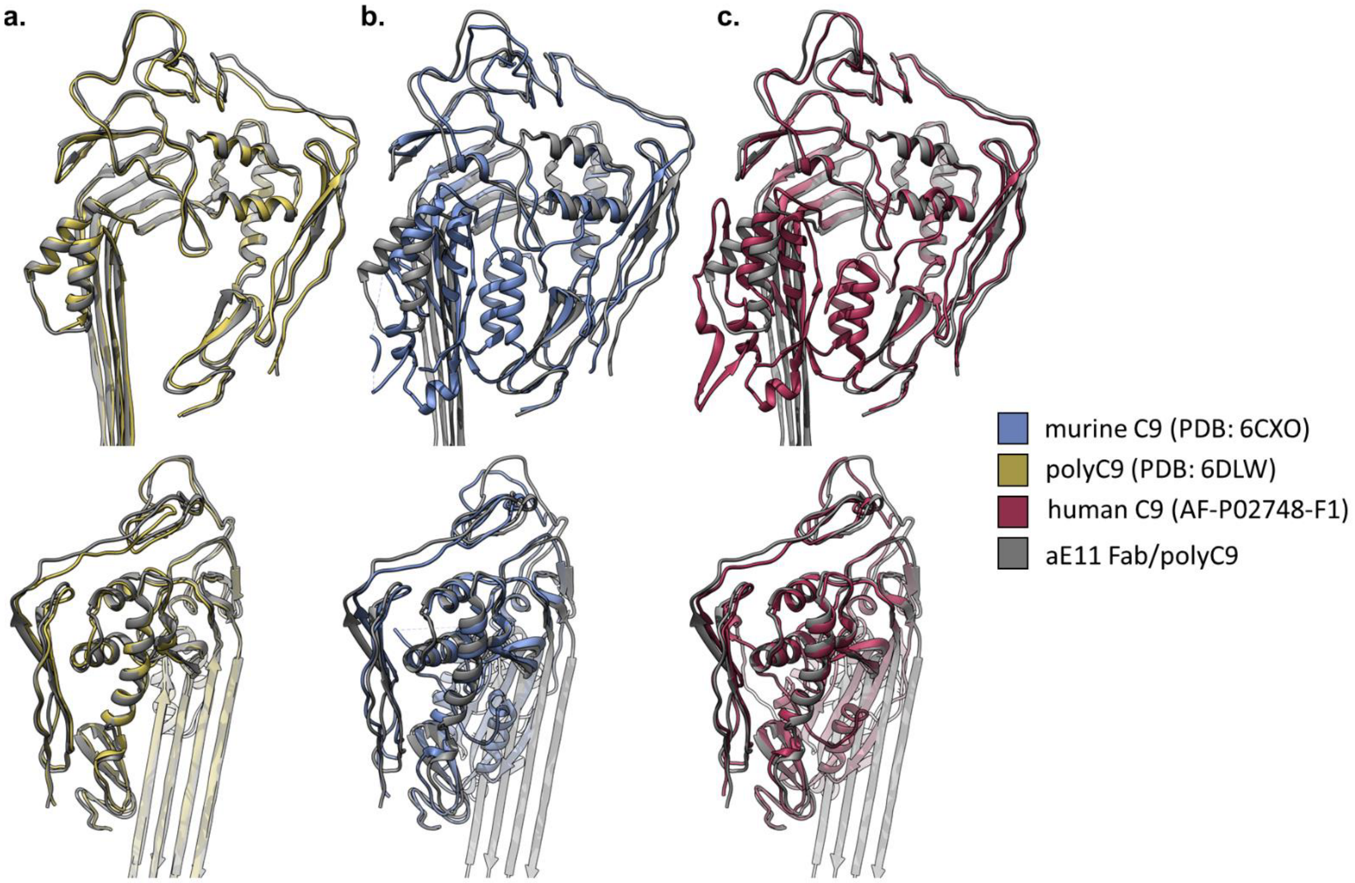

Supp Figure 2. Structural superposition of C9 shows no major conformational differences in the aE11 binding region. a. Comparison of polyC9 without (yellow) and with (grey) bound aE11. **b.** Murine C9 crystal structure (PDB 6CXO) superimposed on aE11-Fab bound polyC9 shows minor shifts in the thrombospondin and low-density lipoprotein receptor A (TSP/LDLRA) region of C9. **c.** AlphaFold model of monomeric human C9 superimposed on aE11-Fab bound polyC9 is consistent with murine C9 crystal structure and shows little to no differences in conformation[1].

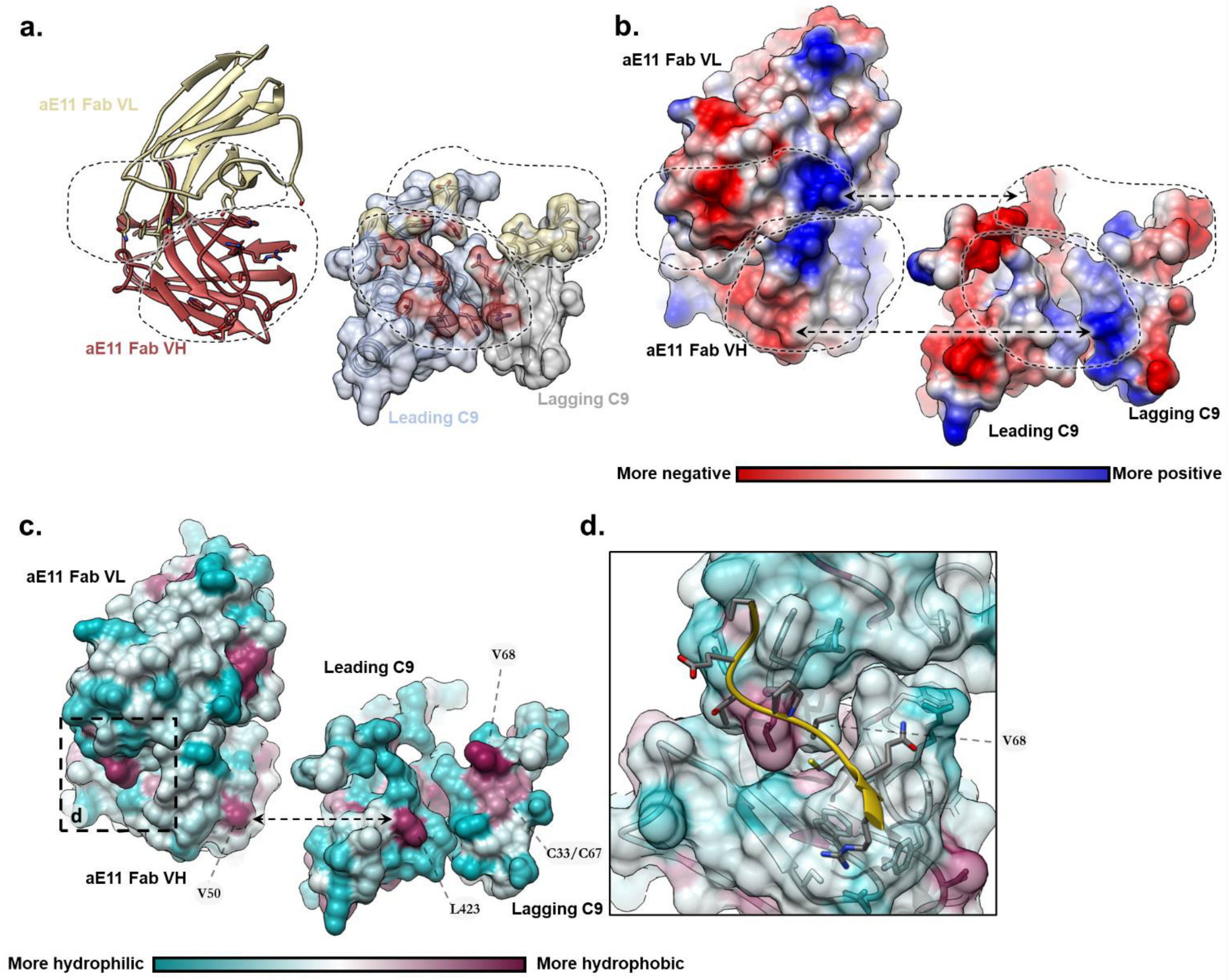

Supp Figure 3. Structural analysis of aE11-Fab/polyC9 interface. a. Cartoon and surface representations of the aE11-CDRs and the C9 epitope respectively. The footprint of the aE11-CDRs is shaded on the C9 epitope by the colour of the aE11 chains. **b.** Identical views from (a) with coulombic surface colouring. Distinct charge complementarity is present between the aE11-CDR and the C9 epitope. Two dominantly charged interfaces are observed. **c.** Identical views from (a) with surface hydrophobicity colouring. Two hydrophobic regions are present on the C9 epitope predominantly defined by L423 and V68. Double headed arrow indicates interacting residues. **d.** Boxed region from (c) showing a small region of the lagging C9 TSP domain (yellow cartoon; contributing to the hydrophobic surface region shown in [c.]) that is sheltered by the aE11 CDR H3 and L3 groove (surface rendering coloured by hydrophobicity).

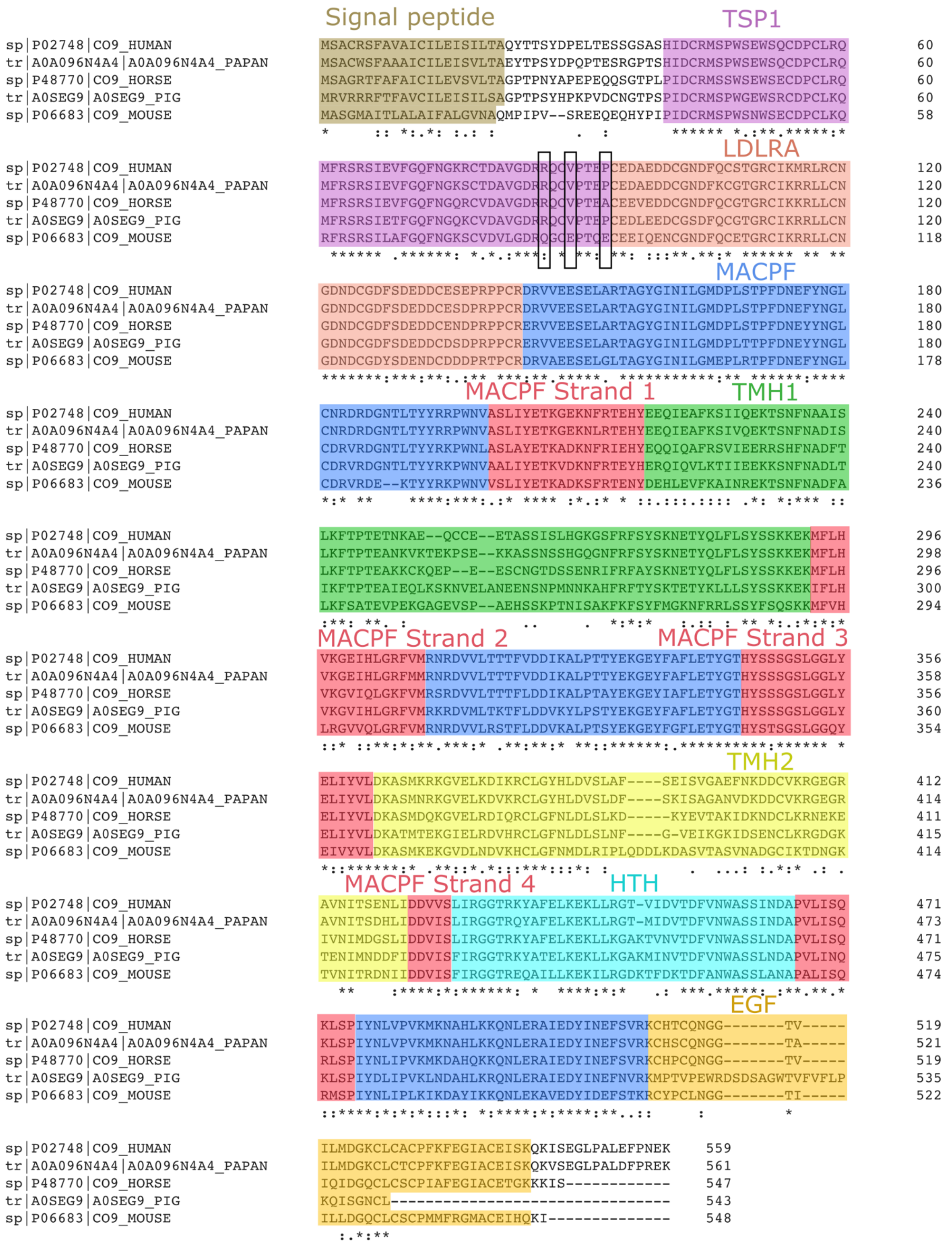

Supp Figure 4. Multiple sequence alignment of C9 homologs. Alignment of human, baboon (*Papio anubis*), horse, pig and mouse complement C9. Horizontal box shows signal peptide which is removed during secretion. Vertical boxes show key aE11 contact residues that disrupt binding upon mutation. The residue numbering is shown for the full open reading frame of the C9 gene. TSP1 domain (purple), LDLRA (salmon pink), body of MACPF domain (blue), conserved core β-sheet of MACPF domain (red), TMH1 region of MACPF domain (green), TMH2 region of MACPF Domain (yellow), HTH region of MACPF domain (cyan), and EGF-like domain (orange).

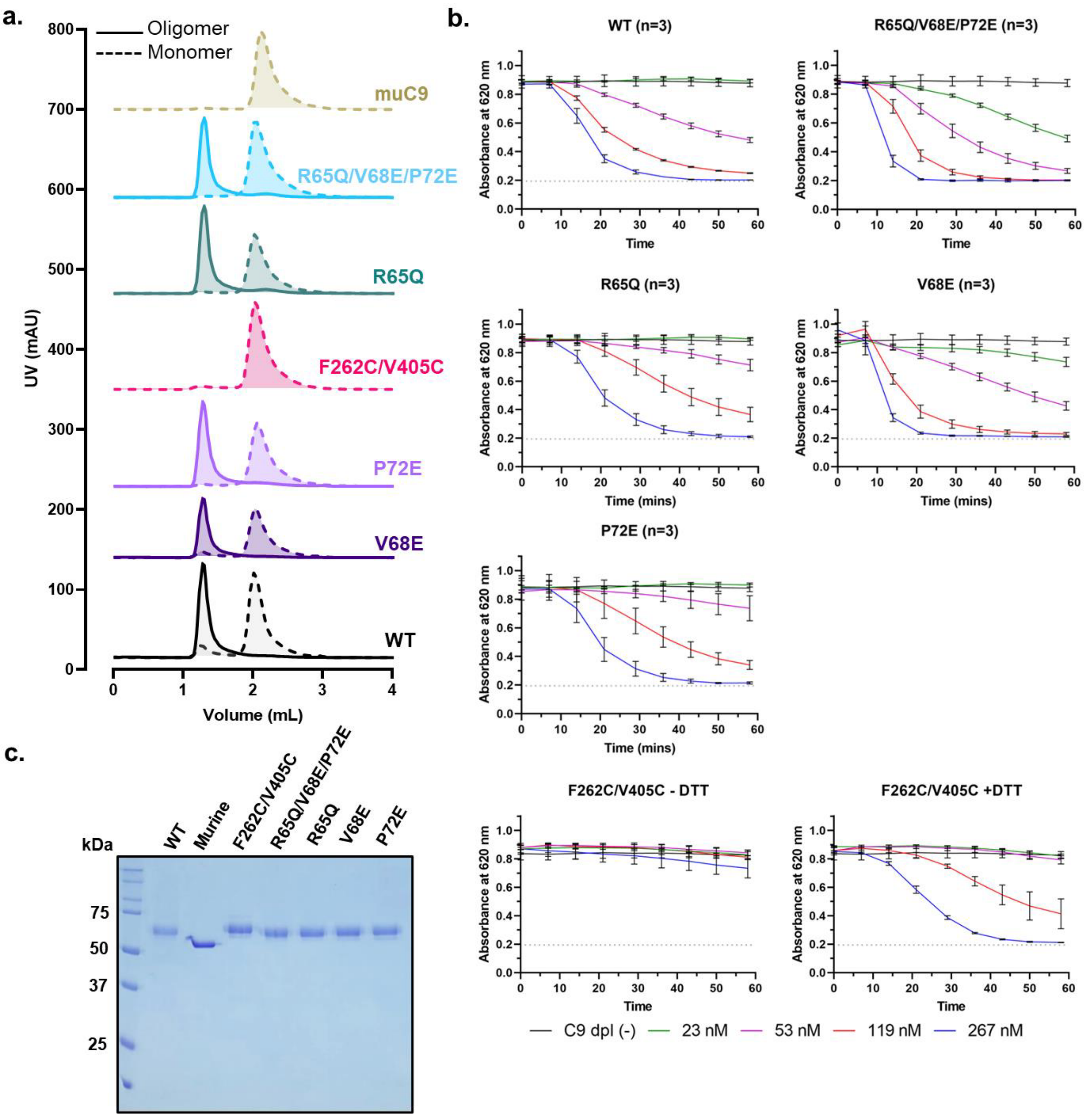

Supp Figure 5. Functional comparison of C9 variants (monomeric *versus* polyC9). **a.** Analytical size-exclusion chromatography of wild-type and variant forms of monomeric and oligomeric C9. Oligomeric C9 elutes in the void volume (~1.2 mL), while monomeric C9 (~2.1 mL) is resolved by the column. **b.** Red blood cell haemolytic time-course assays of MAC assembly with C9 variants (n=3). Concentration-series of wild-type and variant forms of C9 are shown as individual curves. Every tenth data point is shown. All plots are shown as averages, with error bars presented as the standard error of the mean (SEM). The absorbance at 30 minutes was taken for each concentration of C9 to determine the effective half-maximal concentration. The additional experiment of reducing and non-reducing conditions was conducted for the disulphide trapped C9 (F262C/V405C) variant to illustrate inactivity and activity respectively (+DTT is 1 mM final DTT concentration). **c.** Non-reducing SDS-PAGE (12% w/w) of purified recombinant C9 constructs.

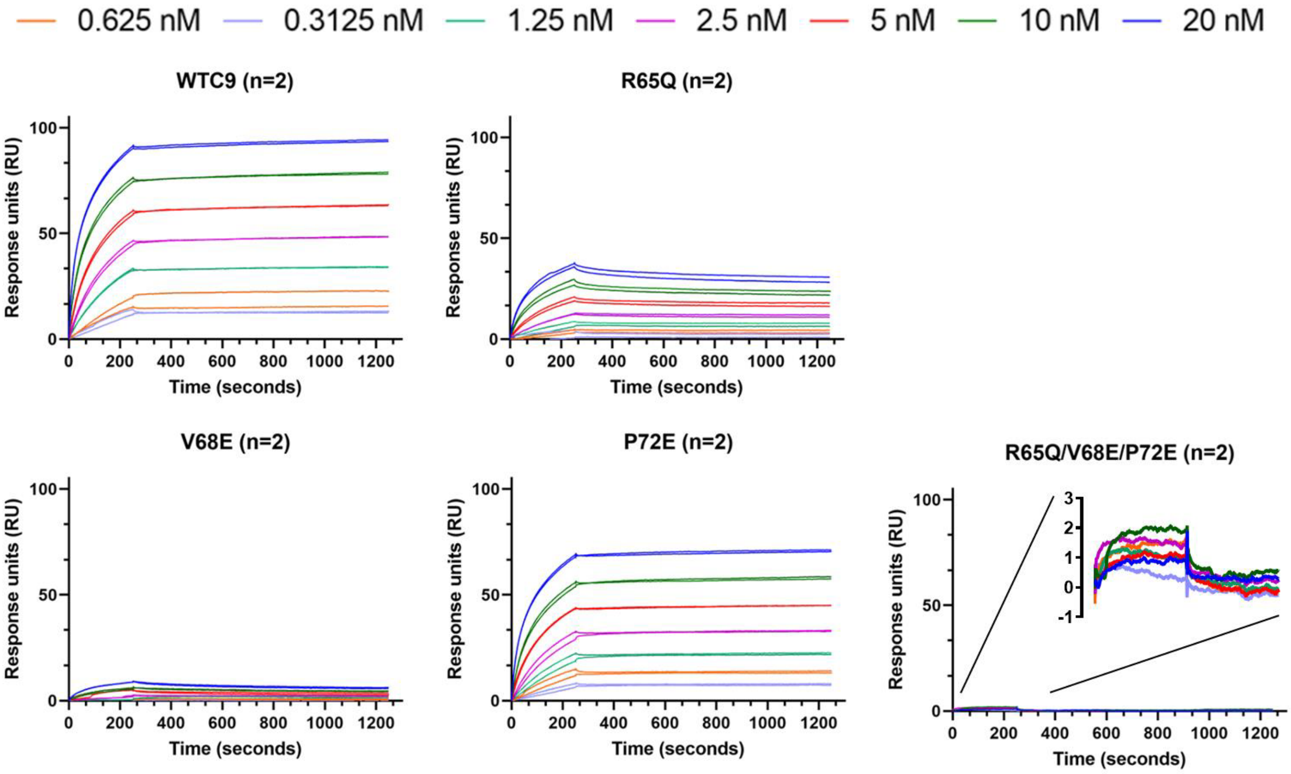

Supp Figure 6. Raw sensorgrams of polymeric C9 and aE11 interactions. Binding of different oligomeric C9 variants to full-length aE11 IgG as measured by surface plasmon resonance. PolyC9 reactions were performed overnight prior to analytical size exclusion. Concentrations of polyC9 are outlined in the legend above the sensorgrams.

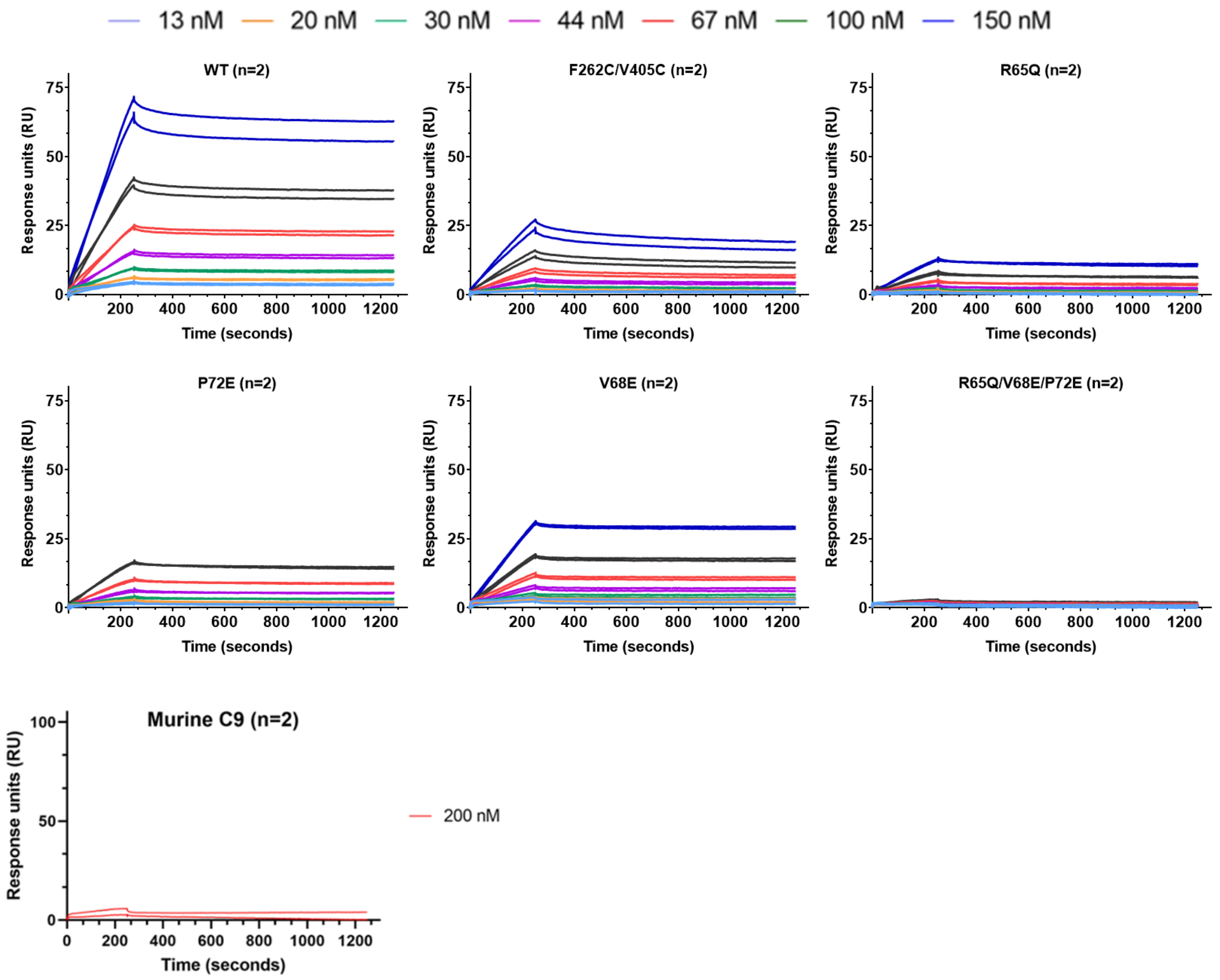

Supp Figure 7. Raw sensorgrams of monomeric C9 and aE11 interactions. Binding of different C9 variants to full-length aE11 IgG as measured by surface plasmon resonance. Regeneration steps were performed between each subsequent concentration, as well as between each variant. Concentrations of the C9 analytes at each injection round are coloured consistently between graphs (values shown in legend key). Monomeric murine C9 was applied at 200 nM as a control for non-specific binding.

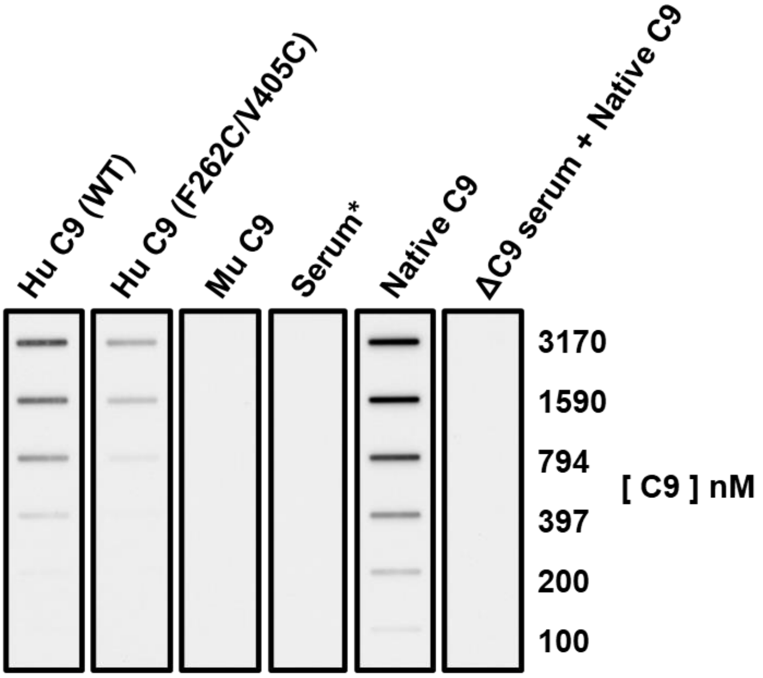

Supp Figure 8. Slot immunoblot of aE11 binding to monomeric purified C9 or human serum. A concentration series of human (Hu) and murine (Mu) C9 variants in the monomeric state, serum and C9-depleted serum that was supplemented with recombinant C9 detected by aE11 IgG in a slot immunoblot assay. Native C9 was diluted into C9 depleted serum to a final concentration of 3170 nM, serial dilution was conducted with C9 depleted serum (Comp Tech). (*) Since C9 concentrations in serum is unknown, it was initially added undiluted and then serially diluted 2-fold; the approximate physiological concentration of monomeric C9 in neat plasma is ~900±200 nM[2,3].

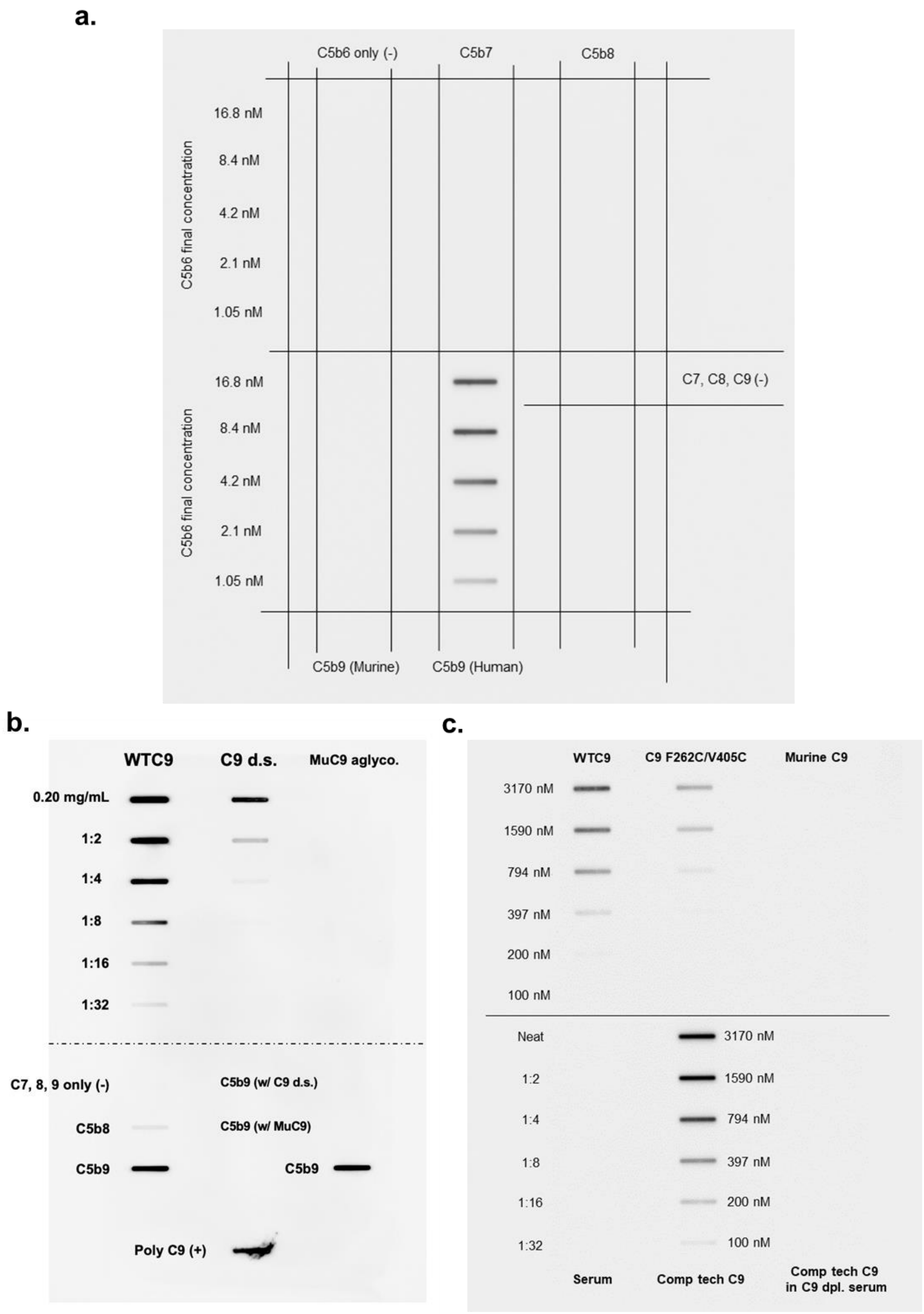

Supp Figure 9. Uncropped immunoblots. Corresponding uncropped, unmodified, immunoblots for **a.** Fig 2f, **b.** 4a, and **c.** Supplementary Figure 8.

References

1. Jumper, J. et al. Highly accurate protein structure prediction with AlphaFold. Nature 596, 583-589 (2021).

2. Morgan, P. Chapter 22 - C9. in The Complement FactsBook (Second Edition) (eds. Barnum, S. & Schein, T.) 231-237 (Academic Press, 2018).

3. Oleesky, D.A. et al. Complement component C9 in Graves' disease. Clin Endocrinol (Oxf) 25, 623-32 (1986).

Supplementary Table 1. Residue level interactions and contact tables of aE11/C9 neoepitope interface.

INTERFACE RESIDUES						
**Residue**	**Number**	**ASA**	**BSA**	**Δ^{i}G**	**Chain**	**HDSC* bonds**
Leading C9 and aE11 heavy chain						
PRO	35	37.35	0.33	0.01	C9 lead	
THR	70	139.08	58.56	0.38		
GLU	71	101.10	74.51	-0.39		HS
PRO	72	115.73	10.69	0.17		
GLU	74	132.91	23.24	-0.22		
ALA	76	68.01	29.03	0.26		H
GLU	77	87.00	7.08	0.11		
ASP	78	106.48	3.34	-0.04		
THR	276	58.04	18.80	0.29		
THR	277	91.12	26.28	0.42		
LYS	419	103.86	37.88	0.40		
ASN	420	69.06	48.01	-0.28		
HIS	422	127.13	33.92	0.18		
LEU	423	88.16	58.59	0.93		
GLN	20	193.65	48.01	-0.21	aE11 heavy chain	
VAL	21	40.61	20.33	0.33		
GLY	45	56.38	7.33	-0.07		
PHE	46	35.37	3.78	0.06		
SER	47	61.93	5.53	-0.06		
THR	49	77.31	8.18	0.02		
VAL	50	85.26	66.44	0.98		
TYR	51	57.60	11.85	-0.06		
ASP	73	101.35	7.82	-0.02		
ARG	116	34.42	12.38	-0.25		HS
ARG	118	100.60	56.85	-1.55		H
SER	119	43.24	32.64	0.52		
TYR	120	103.95	89.30	0.92		H
TRP	126	161.17	39.56	0.23		
TYR	129	106.17	25.38	-0.17		
Lagging C9 and aE11 heavy chain						
TRP	30	70.86	8.83	0.00	C9 lag	
ARG	38	127.95	7.28	-0.08		
MET	40	31.50	7.20	0.12		
ARG	65	116.74	74.00	-1.2		HS
GLN	66	158.03	44.31	-0.14		
CYS	67	22.02	12.72	0.16		
VAL	68	122.22	42.01	0.67		
TRP	71	62.91	51.86	0.83	aE11 heavy chain	
ASP	73	101.35	15.76	-0.14		HS
SER	75	48.72	15.24	-0.10		
TYR	120	103.95	14.65	0.01		
GLY	121	10.26	0.98	-0.01		
GLY	122	48.24	43.20	-0.12		

SER	123	72.87	35.46	0.28		
SER	124	89.49	16.92	-0.10		
Lagging C9 and aE11 light chain						
ARG	38	127.95	40.78	-1.00	C9 lag	H
VAL	68	122.22	68.43	0.85		
PRO	69	19.72	17.71	-0.07		H
THR	70	145.06	43.14	0.29		
GLU	71	109.20	1.87	0.03		
PRO	72	114.86	52.78	0.82		
HIS	46	102.42	20.40	0.12	aE11 light chain	
GLY	110	32.51	2.99	-0.03		
ASN	111	52.27	38.91	-0.51		H
TYR	112	110.22	86.57	1.04		
LEU	113	148.26	70.11	0.63		H
PRO	114	83.02	1.17	0.02		
TYR	115	126.98	12.39	-0.07		
Leading C9 and aE11 light chain						
PRO	72	115.73	30.80	0.49	C9 lead	
GLU	74	132.91	56.22	0.31		H
ALA	76	68.01	30.61	0.49		
GLU	77	87.00	6.08	-0.01		
ASP	78	106.48	29.63	0.47		
ASP	79	122.52	44.37	-0.43		H
TYR	51	91.78	18.01	-0.21	aE11 light chain	
TYR	68	77.66	41.50	0.16		H
TYR	69	103.32	56.61	0.18		H
ARG	72	134.89	28.54	-0.49		
HIS	74	76.29	16.48	0.17		
SER	75	98.62	39.41	0.22		

RESIDUE CONTACTS									
**Chain**	**Residue**	**Atom**	**Number**	**Distance**	**Chain**	**Residue**	**Atom**	**Number**	**Type**
Leading C9 and aE11 heavy chain									
C9 lead	HIS	ND1	422	3.68	aE11 H	TYR	OH	120	H
C9 lead	GLU	OE1	71	3.64	aE11 H	ARG	NH1	116	H
C9 lead	ALA	O	76	3.10	aE11 H	ARG	NH2	118	H
C9 lead	ASN	OD1	420	3.53	aE11 H	TYR	N	120	H
C9 lead	GLU	OE1	71	3.64	aE11 H	ARG	NH1	116	S
Lagging C9 and aE11 heavy chain									
C9 lag	ARG	NH1	65	3.62	aE11 H	ASP	OD2	73	H
C9 lag	ARG	NH1	65	3.62	aE11 H	ASP	OD2	73	S
Lagging C9 and aE11 light chain									
C9 lag	ARG	NH2	38	3.17	aE11 L	ASN	OD1	111	H
C9 lag	PRO	O	69	3.66	aE11 L	LEU	N	113	H
Leading C9 and aE11 light chain									
C9 lead	ASP	N	79	3.19	aE11 L	TYR	OH	69	H
C9 lead	GLU	OE2	74	2.98	aE11 L	TYR	OH	68	H

Supplementary table 2. Oligonucleotide primer sequences for site directed mutagenesis

**Mutation**	**Sequence 5'→3'** (mutations in **bold**)
R65Q_f	CGCTGTGGGAGACAGA**CAA**CAGTGTGTGCCCACAGAGCCC
R65Q_r	CTGTGGGCACACACTG**TTG**TCTGTCTCCCACAGCGTCGG
V68E_f	GAGACAGACGACAGTGT**GAG** CCCACAGAGCCCTGTGAGG
V68E_r	CAGGGCTCTGTGGG**CTC** ACACTGTCGTCTGTCTCCCAC
P72E_f	GTGTGCCCACAGAG**GAA**TGTGAGGATGCTGAGGATGACTGCGG
P72E_r	CCTCAGCATCCTCACA**TTC** CTCTGTGGGCACACACTGTCG
R65Q/V68E/P72E_f	GA**CAA**CAGTGT**GAG**CCCACAGAG**GAA**TGTGAGGATGCTGAGG
R65Q/V68E/P72E_r	CA**TTC**CTCTGTGGG**CTC**ACACTG**TTG**TCTGTCTCCCACAGCG
F262C_f	ACTTACCAACTA**TGT**TTGTCATATTCTTCAAAG
F262C_r	GAAGAATATGACAA**ACA**TAGTTGGTAAGTTTC
V405C_f	CATAGATGATGTT**TGT**TCACTCATAAGAGGTGG
V405C_r	CTCTTATGAGTGA**ACA**AACATCATCTATGAG

Note primers correspond to codon optimised synthetic gene and may not necessarily reflect the sequence of the endogenous C9 gene.
