# Supplementary material for: The neoepitope of the complement C5b-9 Membrane Attack Complex is formed by proximity of adjacent ancillary regions of C9
Source: Commun Biol. 2023 Jan 13;6:42. doi: 10.1038/s42003-023-04431-y (PMC9838529; doi:10.1038/s42003-023-04431-y)
Supplement: Supplementary file 3 — Description of Additional Supplementary Files [file 42003_2023_4431_MOESM3_ESM.pdf]

Description of Additional Supplementary Files

File name: Supplementary Data 1
Description: All source data for figures 1, 2, 3, 4 and 5.
