# Supplementary material for: The neoepitope of the complement C5b-9 Membrane Attack Complex is formed by proximity of adjacent ancillary regions of C9
Source: Commun Biol. 2023 Jan 13;6:42. doi: 10.1038/s42003-023-04431-y (PMC9838529; doi:10.1038/s42003-023-04431-y)
Supplement: Supplementary file 5 — Reporting Summary [file 42003_2023_4431_MOESM5_ESM.pdf]

nature portfolio

Corresponding author(s): Bradley Spicer & Michelle Dunstone

Last updated by author(s): Jul 4, 2022

Reporting Summary

Nature Portfolio wishes to improve the reproducibility of the work that we publish. This form provides structure for consistency and transparency in reporting. For further information on Nature Portfolio policies, see our Editorial Policies and the Editorial Policy Checklist.

Statistics

For all statistical analyses, confirm that the following items are present in the figure legend, table legend, main text, or Methods section.

n/a	Confirmed	
☐	☒	The exact sample size (*n*) for each experimental group/condition, given as a discrete number and unit of measurement
☐	☒	A statement on whether measurements were taken from distinct samples or whether the same sample was measured repeatedly
☒	☐	The statistical test(s) used AND whether they are one- or two-sided<br>*Only common tests should be described solely by name; describe more complex techniques in the Methods section.*
☒	☐	A description of all covariates tested
☒	☐	A description of any assumptions or corrections, such as tests of normality and adjustment for multiple comparisons
☒	☐	A full description of the statistical parameters including central tendency (e.g. means) or other basic estimates (e.g. regression coefficient) AND variation (e.g. standard deviation) or associated estimates of uncertainty (e.g. confidence intervals)
☒	☐	For null hypothesis testing, the test statistic (e.g. *F*, *t*, *r*) with confidence intervals, effect sizes, degrees of freedom and *P* value noted<br>*Give P values as exact values whenever suitable.*
☒	☐	For Bayesian analysis, information on the choice of priors and Markov chain Monte Carlo settings
☒	☐	For hierarchical and complex designs, identification of the appropriate level for tests and full reporting of outcomes
☒	☐	Estimates of effect sizes (e.g. Cohen's *d*, Pearson's *r*), indicating how they were calculated

Our web collection on statistics for biologists contains articles on many of the points above.

Software and code

Policy information about availability of computer code

Data collection	The CryoEM data was collected with SerialEM or EPU. NanoDSF was collected using PR.ThermControl. Mass photometry was collected with Refeyn AcquireMP v2.5
Data analysis	CryoEM analysis: IMOD, MotionCor2 (v.1.3.0), CTFFIND (v4.1.13), RELION (v3.2), cryoSPARC (v3.3.1), pyEM, localised_reconstruction.py, SWISS-MODEL, UCSF Chimera (v1.14), UCSF ChimeraX (v1.3), Coot (v0.9.2), ISOLDE (v.1.0), PHENIX (v1.17), MolProbity, wwPDB One Dep. Antibody V-gene and allele retrieval assisted by IMGT. AUC analysis: SEDNTERP and SEDFIT. SPR was evaluated with BIAevaluation software. Mass photometry Refeyn DiscoverMP v2.5 and GraphPad Prism.

For manuscripts utilizing custom algorithms or software that are central to the research but not yet described in published literature, software must be made available to editors and reviewers. We strongly encourage code deposition in a community repository (e.g. GitHub). See the Nature Portfolio guidelines for submitting code & software for further information.

Data

Policy information about availability of data

All manuscripts must include a data availability statement. This statement should provide the following information, where applicable:

- Accession codes, unique identifiers, or web links for publicly available datasets
- A description of any restrictions on data availability
- For clinical datasets or third party data, please ensure that the statement adheres to our policy

The Cryo-EM map is deposited in the Electron Microscopy Data Bank (EMDB) EMD-27385. The atomic coordinates of polyC9-aE11 are available from the RCSB Protein Data Bank under the accession code 8DE6. Additional information and data are made available upon reasonable request from the corresponding authors.

Human research participants

Policy information about studies involving human research participants and Sex and Gender in Research.

Reporting on sex and gender	N/A
Population characteristics	N/A
Recruitment	N/A
Ethics oversight	N/A

Note that full information on the approval of the study protocol must also be provided in the manuscript.

Field-specific reporting

Please select the one below that is the best fit for your research. If you are not sure, read the appropriate sections before making your selection.

☒ Life sciences ☐ Behavioural & social sciences ☐ Ecological, evolutionary & environmental sciences

For a reference copy of the document with all sections, see nature.com/documents/nr-reporting-summary-flat.pdf

Life sciences study design

All studies must disclose on these points even when the disclosure is negative.

Sample size	All experiments in this study were performed in vitro. Individual mutants, and functional assays and measurements were performed with a replica of n=3.
Data exclusions	Outliers were excluded from haemolytic assays in events where air bubbles or plate reader errors caused large spikes. Otherwise no data were excluded.
Replication	All attempts at reproducing results were successful. Experiments were performed multiple times.
Randomization	Randomization was not performed in this study. There were no clinical or cohort studies. All experiments were performed in vitro.
Blinding	Blinding was not performed in this study. The general methodology applied in this study of assessing MAC function are well documented in the literature.

Reporting for specific materials, systems and methods

We require information from authors about some types of materials, experimental systems and methods used in many studies. Here, indicate whether each material, system or method listed is relevant to your study. If you are not sure if a list item applies to your research, read the appropriate section before selecting a response.

Materials & experimental systems

n/a	Involved in the study
☐	☒ Antibodies
☐	☒ Eukaryotic cell lines
☒	☐ Palaeontology and archaeology
☐	☒ Animals and other organisms
☒	☐ Clinical data
☒	☐ Dual use research of concern

Methods

n/a	Involved in the study
☒	☐ ChIP-seq
☒	☐ Flow cytometry
☒	☐ MRI-based neuroimaging

Antibodies

Antibodies used: Anti-MAC antibody aE11, source Monoclonal Mouse IgG2a kappa

Validation: While aE11 is commercially available, the source of antibody used in this study was provided by co-author Mollnes and Lau. The specificity for aE11 recognition to a neoepitope region of the MAC has been studied extensively.

T E Mollnes, T Lea, M Harboe, and J Tschopp. 1985. Scandinavian Journal of Immunology 22(2):183-195
T E Mollnes, T Lea, S S Froland, and M Harboe. 1985. Scandinavian Journal of Immunology 22(2):197-202
M Harboe, E B Thorgersen, and T E Mollnes. 2011. Adv Drug Deliv Rev 63(12):976-987
P Garred, A J Tenner, and T E Mollnes. 2021. Pharmacol Rev 73(2):792-827

Eukaryotic cell lines

Policy information about cell lines and Sex and Gender in Research

Cell line source(s): Expi293F cells (Thermo Fisher Scientific) Cat no: A14528

Authentication: The cells were not authenticated in this study.

Mycoplasma contamination: The cells were not tested for mycoplasma contamination.

Commonly misidentified lines (See ICLAC register): No commonly misidentified cell lines were used in this study.

Animals and other research organisms

Policy information about studies involving animals; ARRIVE guidelines recommended for reporting animal research, and Sex and Gender in Research

Laboratory animals: Only in vitro experiments were performed in this study. In some instances these involved the use of animal derived products that were obtained commercially (e.g. blood and antibodies). No special animal ethics approval are required for these tests.

Wild animals: This study did not involve wild animals

Reporting on sex: As no animals were used in this study, no reporting on sex was performed.

Field-collected samples: This study did not involve samples collected from the field.

Ethics oversight: No ethics oversight was conducted as all experiments were in vitro.

Note that full information on the approval of the study protocol must also be provided in the manuscript.
